# Establishing a national biosafety and biosecurity agency for the United States

**DOI:** 10.3389/fbioe.2024.1474120

**Published:** 2024-10-17

**Authors:** David R. Gillum, Rebecca Moritz, Gregory D. Koblentz

**Affiliations:** ^1^ Research and Innovation, University of Nevada, Reno, NV, United States; ^2^ School for the Future of Innovation and Society, Arizona State University, Tempe, AZ, United States; ^3^ Tutela Strategies, LLC, Reno, NV, United States; ^4^ Office of the Vice President for Research, Colorado State University, Fort Collins, CO, United States; ^5^ Schar School of Policy and Government, George Mason University, Arlington, VA, United States

**Keywords:** biosafety, biosecurity, legislation, biotechnology regulation, incident response, unified biosafety agency, international collaboration

## Abstract

The rapid advancement of biological research and biotechnology requires a novel and robust regulatory agency to ensure uniform biosafety and biosecurity governance in the United States. The current fragmented regulatory landscape needs to be refocused to address the complexities of modern biological research, including risks associated with accidental, inadvertent, and deliberate biological incidents. An independent government agency, which we call the National Biosafety and Biosecurity Agency (NBBA), that is devoted to biosafety and biosecurity could effectively address these challenges. The NBBA would consolidate various regulatory functions, streamline processes, and enhance oversight. This oversight would encompass life sciences research in the United States, regardless of the source of funding or level of classification. The agency could also contribute to the bioeconomy by streamlining requirements to safeguard public health and the environment while fostering scientific and commercial progress. The proposed agency would govern high-risk biological pathogens, manage the Federal Select Agent Program, enforce policies related to dual use research of concern, pathogens with enhanced pandemic potential, and nucleic acid synthesis screening, administer regulations on the use and care of laboratory animals, as well as regulate other relevant biosafety and biosecurity activities. The goal would be to provide one-stop shopping for the biomedical research and biotechnology sectors subject to oversight by the Federal government. To ensure leadership in global biosafety and biosecurity, the agency’s mission would include international collaboration, applied research, education, workforce development, and coordination with national security initiatives. Creating an agency like the NBBA will be politically challenging but presenting a comprehensive vision and engaging stakeholders early and frequently, and being transparent in the process, will be essential for garnering support. Creating a unified biosafety and biosecurity governance system in the United States will ensure the safe and secure advancement of biological research while sustaining innovation and maintaining international competitiveness.

## 1 Introduction

Forming a single agency, which we call the National Biosafety and Biosecurity Agency (NBBA), dedicated to biosafety and biosecurity is necessary to mitigate biological risks both domestically and internationally (Ritterson, et al., 2022; DiEuliis and Giordano, 2022; Koblentz and Casagrande, 2023). The biological risk landscape is rapidly evolving and presents significant new challenges to preventing the accidental, inadvertent, or malicious misuse of biology (Lentzos et al., 2022). The U.S. biological risk management system, characterized by fragmented oversight and varying levels of authority across agencies, struggles to effectively address the complexities of modern life sciences research (Lin, 2010; Kelle, 2013; Lim et al., 2021; Le Duc and Weaver, 2024). This disjointed approach often results in challenges with coordination and responsiveness, as noted by Gillum et al. (2022). Agencies often fail to coordinate effectively, resulting in disagreements or unilateral changes that impact other agencies without proper consultation, alignment of goals, or collaborative integration of efforts. These agencies also lack the agility and responsiveness required for novel biotechnologies, requiring a creative, outside-the-box solution (Miller and Bennett, 2008; Koblentz, 2014; Evans et al., 2020). Many policies apply only to Federally funded research, despite the significant growth in pathogen research and biotechnology innovation in the private sector (Greene, et al., 2023; and Lentzos et al., 2022). The fortuitous discovery of an illegal biotech company in Reedley, California that was storing human pathogens without proper biosafety is a cautionary tale (Greene et al., 2023). Given the growing role of the private sector in conducting biotechnology research and the growth of the bioeconomy, the exclusion of almost all of the work of the private sector from dual-use research oversight is an increasingly large loophole.

The current, lively debates surrounding the origins of the SARS-CoV-2 virus responsible for the COVID-19 pandemic, and concerns about research that could potentially create a future pandemic pathogen highlight the urgent need for a unified regulatory approach (Bory et al., 2021; Nie, 2020). This new agency would be responsible for addressing the safety, security, and ethical dilemmas regarding whether certain types of biological research should take place and under what conditions (Atlas and Dando, 2006; Miller and Selgelid, 2007; Kuhlau et al., 2008). The dramatic growth in the bioeconomy and the proliferation of new biology-based products is also creating new challenges (Hodgson et al., 2022; Warmbrod et al., 2020). There is an ongoing need to balance commercial interests, such as new biotechnology companies navigating the complex and often inconsistent regulations for biological production and manufacturing, with safety and security (Vallas and Kleinman, 2008; Attal-Juncqua et al., 2023; The White House, 2022). A government body singularly devoted to biosafety and biosecurity would unify disparate regulatory efforts, simplify procedures, and improve oversight, thereby protecting public health and the environment while supporting scientific advancement and technological innovation.

Although the motivation for proposing a new agency for biosafety and biosecurity is frequently framed around traditional risks such as bioterrorism and laboratory incidents, it is essential to have a flexible system that can consider emerging risks, such as synthetic biology, genome editing, automated labs, and artificial intelligence. In addition, research security, particularly in the life sciences, has become a national-level concern due to geopolitics, control of intellectual property rights, and the risks posed by adversarial nation state actors (You, 2017; Richardson, et al., 2019; The White House, 2021). Without acknowledging and incorporating these contemporary risks, stakeholders may fail to appreciate the necessity for a significant transformation in the regulatory framework for biosafety and biosecurity (Beck, 1992; Wynne, 1992; Eisner, 2000). It is essential not only to justify the need for substantial change, but also to clearly articulate the specific motivations and threats posed by both traditional and emerging risks (Vogel, 2012; Gronvall, 2013; National Academies of Sciences, Engineering, and Medicine, 2017).

Despite the considerable political and operational obstacles faced by the creation of such a body, it is the goal of this article to provide a thorough analysis and concrete recommendations for how this oversight institution could operate. The new agency must possess the flexibility necessary to navigate within shifting political landscapes as well as address emergent biotechnology issues over time. Initiating open dialogue with interested parties, committing to transparency in operations, and extending outreach to professional communities, while receiving input from the public, private sector, and non-governmental organizations, will be crucial to acquire the necessary political support and ensure the new agency is inclusive and addresses concerns from multiple viewpoints (Epstein, 1995; Jasanoff, 2006; Kanabrocki, 2011; Kaplan et al., 2021).

The creation of a national biosafety and biosecurity agency will mark a noteworthy progression in protecting against potential risks associated with the life sciences (Atlas and Reppy, 2005). By unifying control and oversight, optimizing regulations, advancing evidence-based biosafety and biosecurity policies and practices, promoting international cooperation, and elevating education and workplace development, the NBBA would bolster national and global biosafety and biosecurity measures. Adopting this approach will help ensure the prudent progression of life sciences research and uphold the United States bioeconomy and global competitive edge, while appropriately navigating risks and benefits through an effective and evolving regulatory structure. This article provides the most detailed set of recommendations yet published on the creation of a national, unified governance system for biosafety and biosecurity in the United States.

The proposed mission, scope, responsibilities, and authorities of the NBBA are designed to meet the five key elements of effective oversight identified by the Government Accountability Organization (GAO) (United States Government Accountability Office, 2017).

- Independence: The organization conducting oversight should be structurally distinct and separate from the entities it oversees.
- Ability to perform reviews: The organization should have the access and working knowledge necessary to review compliance with requirements.
- Technical expertise: The organization should have sufficient staff with the expertise to perform sound safety and security assessments.
- Transparency: The organization should provide access to key information, as applicable, to those most affected by operations.
- Enforcement authority: The organization should have clear and sufficient authority to require that entities achieve compliance with requirements

## 2 Creation of a unified biosafety and biosecurity management agency

### 2.1 Regulatory framework

The regulatory framework for the proposed NBBA should be comprehensive and consolidate existing governance structures and mechanisms into a single entity to eliminate redundancies and streamline processes (Gillum et al., 2023). This “one-stop shop” approach would provide clear and consistent guidelines for institutions, researchers, safety professionals, and the public, ensuring efficient and effective compliance while limiting oversight gaps. The NBBA would need to possess robust regulatory authority to verify compliance with biosafety and biosecurity requirements and take enforcement actions when necessary. Table 1 provides examples of existing governmental authorities involving biosafety and biosecurity. To maintain integrity and avoid conflicts of interest, the agency should be appropriately funded to ensure that it can operate independently and not be associated with a life science research funding agency.

**TABLE 1:** Examples of existing biosafety and biosecurity regulatory authorities.

Existing biosafety and biosecurity governance mechanism	Regulatory authority
Biological Transport Regulations	49 CFR 173.132–134
CDC Import Permit Program	Public Health Service Act, 42 U.S.C. § 264 (Regulatory authority for the program is given to the Secretary of Health and Human Services), 42 CFR Part 71, and 42 CFR Part 73
Environmental Impact Assessments for Biological Materials	National Environmental Policy Act (NEPA) Regulations, 40 CFR Parts 1500-1508
EPA Biotechnology Regulations	Federal Insecticide Fungicide and Rodenticide Act (7 U.S.C. § 136 et seq.), Federal Food Drug and Cosmetic Act (21 U.S.C. § 301 et seq.), and Toxic Substances Control Act (15 U.S.C. § 2601 et seq.), 40 CFR Part 725
NIH Guidelines for Research Involving Recombinant or Synthetic Nucleic Acid Molecules	Federal Register, Vol. 38, No. 221 (16 November 1973); Federal Register, Vol. 39, No. 165 (23 August 1974) (Binding only on federally-funded activities)
OSHA Bloodborne Pathogens Standard	29 CFR 1910.1030
OSHA Infectious Disease Rulemaking	In rulemaking process, Federal Register Vol. 78 No. 81 (26 April 2013)
USDA and PHS Care and Use of Laboratory Animals	Animal Welfare Act (7 U.S.C. 2131-2159); Animal Welfare Regulations (9 CFR Parts 1-4); Health Research Extension Act of 1985 (42 U.S.C. § 289d); and Public Health Service Policy on Humane Care and Use of Laboratory Animals (42 CFR Part 93)
HHS Screening Framework Guidance for Providers and Users of Synthetic Nucleic Acids and OSTP Framework for Nucleic Acid Synthesis Screening	Executive Order 14110, Safe, Secure, and Trustworthy Development and Use of Artificial Intelligence (30 October 2023), Non-binding except for use of Federal funds
Select Agent Regulations	7 C.F.R. Part 331: Agriculture; 9 C.F.R. Part 121: Animals and Animal Products; 42 C.F.R. Part 73: Public Health
United States Government Policy for Oversight of Dual Use Research of Concern (DURC) and Pathogens with Enhanced Pandemic Potential (PEPP)	Terms and conditions of federal funding (currently Section 2315 of the Consolidated Appropriations Act, 2023–42 U.S.C. § 6627) (Binding only on government funds)
USDA Animal and Plant Import Permits	Animal Health Protection Act (7 U.S.C. § 8301-8317); Plant Protection Act (7 U.S.C. §§ 7701-7772 and 7781-7786), 9 CFR Part 94, 9 CFR Part 95, 9 CFR Part 122

Existing regulatory authorities from agencies such as the Centers for Disease Control and Prevention (CDC), Environmental Protection Agency (EPA), National Institutes of Health (NIH), Department of Transportation (DOT), and United States Department of Agriculture (USDA), would need to be transferred to the new agency. This would also entail the transfer of associated resources and programs from these agencies to NBBA. The consolidation of agency regulations and responsibilities may present significant challenges, including legal, logistical, and bureaucratic hurdles. Effective coordination and collaboration will be essential to manage this transition smoothly. In addition, the NBBA will need new legislative authorities and resources to extend biosafety and biosecurity oversight to the private sector as well (Epstein, 2023).

The NBBA would base its regulatory functions on scientifically-based risk assessments supported, where possible, by evidence from existing empirical data and new applied biosafety and biosecurity research. The agency’s scope should be dynamic and respond to evolving data to remain effective. Regulatory enforcement should be stratified, balancing mandatory regulations for the highest-risk operations with guidance and voluntary compliance for lower risk activities, based upon feedback from a cross-functional team of individuals with different backgrounds and experiences to recommend practices that will be put through standard (e.g., Administrative Procedure Act) review-and-comment mechanisms (Vogel, 2013; Quinlan et al., 2016; Kojima et al., 2018). Institutions, researchers, biosafety and biosecurity professionals, and other stakeholders should be provided with tools to make compliance more efficient, fostering an environment where adherence to safety standards is practical, achievable, and even rewarded. The agency would also need to develop and enforce regulations, standards, and policies in a transparent manner with multiple opportunities for input and feedback from the full range of stakeholders. By developing a comprehensive scope and well-defined responsibilities for the agency, the United States will be well positioned to effectively address the complexities of modern biological research and biotechnology development.

### 2.2 Scope and responsibilities

The overarching mission of the proposed agency is to ensure the safe, secure, and responsible conduct of life sciences research and biotechnology development in the United States. To achieve this mission, the proposed agency would have oversight of biosafety, biosecurity, dual-use research, and the care and use of laboratory animals. Biosafety in this context refers to activities like protecting researchers in the field from infectious diseases while collecting samples, protecting workers in laboratories conducting research with biohazardous materials, protecting communities from the accidental release of a biohazard from a laboratory or shipping container, and protecting the environment from the accidental or unapproved release of a genetically modified microorganism. Biosecurity in this context refers to functions such as protecting pathogens, valuable biological materials, and data from unauthorized access or deliberate misuse, including ensuring appropriate physical, personnel, and cyber security measures. Responsible conduct in this context refers to governance measures against unanticipated or inadvertent risks by providing oversight of dual-use research, reviewing proposals for research with pathogens with enhanced pandemic potential, developing safeguards for the synthesis of nucleic acids and benchtop nucleic acid synthesizers, and ensuring that laboratory animals are treated humanely and ethically. This definition of responsible conduct is narrower than the traditional definition which includes research misconduct issues such as allegations of fabrication, falsification, or plagiarism. In support of these functions, the NBBA would conduct and sponsor applied research in biosafety and biosecurity, administer a comprehensive incident reporting and investigation system, provide education, training, and workforce development, lead efforts to share best practices, and conduct risk assessments of emerging technologies that could affect biosafety or biosecurity.

At the regulatory level, NBBA would assume responsibility for ensuring compliance with the Select Agent Regulations (currently administered by the CDC and USDA), the issuance of import permits for biological materials (currently administered by the CDC and USDA), the safe and secure shipping of infectious substances (currently administered by the DOT), and the care and use of laboratory animals (currently overseen by NIH and USDA). In addition, NBBA could be integrated into the U.S. Coordinated Framework for Biotechnology (EPA and USDA, 2017) to ensure the proper representation of biosafety and biosecurity perspectives. While the NBBA would play a role in aligning rules for modified organisms both within the laboratory and their application in the field, the EPA would retain responsibility for environmental concerns from the use of uncontained modified microorganisms. This approach would leverage the strengths of both agencies to ensure comprehensive oversight and regulation. For a list of possible functions, refer to Table 2.

**TABLE 2:** Examples of possible oversight functions for the national biosafety and biosecurity agency.

Function	Description
Biosafety and Biosecurity Compliance Framework	Centralizing biosafety and biosecurity regulations from agencies like CDC, EPA, NIH, and USDA into a single cohesive framework. Developing a compliance monitoring system to ensure adherence to biosafety and biosecurity regulations. Implementing a whistleblower hotline for confidential reporting of non-compliance and safety and security concerns
Dual-Use Research of Concern Oversight	Establishing comprehensive national oversight for DURC to assess risks and benefits, develop risk mitigation plans, and reduce the risk of misuse. Creating guidelines for DURC identification and management for publicly and privately funded research institutions located in the United States or funded by the U.S. government in other countries. Ensuring regular review and monitoring of DURC projects by institutional biosafety committees. Reviewing risks and benefits of proposed research with pathogens with enhanced pandemic potential to determine if proposed research should be conducted and under what conditions. (Legislation is needed for comprehensive oversight of non-federally funded research; without legislation, privately-funded research can only be invited to voluntarily participate.)
Data and Research Security	Implementing robust protocols for the secure storage, handling, and transfer of genetic and biological data. Conducting audits and inspections to ensure data security and compliance with security protocols. Establishing guidelines for data sharing and transparency to facilitate research while ensuring security. Addressing the emerging concept of research security by incorporating best practices from NSF’s Research Security activities and relevant JASON reports. Enhancing these efforts by identifying gaps and areas where NBBA can add unique value, such as developing specialized training programs, creating additional security standards, and facilitating cross-agency collaboration
Pathogens with Enhanced Pandemic Potential (PEPP)	Creating guidelines and oversight mechanisms specifically for PEPP research. Establishing risk mitigation plans and review processes for PEPP projects
Safety and Containment Standards	Establishing and enforcing biological safety levels (e.g., BSL-1 to BSL-4) for containment facilities. Establishing stringent design, construction, and certification standards for high-containment facilities. Maintaining a national registry of all BSL-3 and BSL-4 facilities to monitor and ensure compliance with biosafety and biosecurity standards. Ensuring occupational health and safety standards are appropriate for biological workers. Developing standards to reduce the risk of exposure to infectious diseases during fieldwork. Implementing routine safety audits and inspections to ensure compliance with containment standards
Emerging Technology Oversight	Developing regulations for screening synthetic nucleic acid orders and benchtop nucleic acid synthesis equipment. Maintaining a directory of synthetic nucleic acid providers and manufacturers. Conducting horizon scanning exercises, assessing risks of emerging technologies, and identifying risk mitigation options. Developing and maintaining registries for gene drive research and ecological releases to track and monitor developments in these fields. NBBA should also determine which DNA providers are successfully performing screening and are therefore eligible to sell DNA to users, as mandated by EO 14110
Applied Biosafety and Biosecurity Research	Conducting and funding research projects focused on improving biosafety and biosecurity practices and technologies. Developing and promoting new technologies for enhanced biosafety and biosecurity. Collaborating with academic institutions to advance biosafety and biosecurity research. Providing funding to study occupational health and safety protection measures
Incident Reporting and Investigation	Establishing and maintaining a centralized incident reporting system. Analyzing incident reports to identify trends and improve biosafety and biosecurity protocols. Investigating incidents to determine the cause, identify remedies, and ensure accountability
International and Cross-Sector Collaboration	Coordinating with international bodies to harmonize and improve biosafety and biosecurity standards. Conducting biosafety and biosecurity capacity-building programs for laboratories in partner nations based on international standards (e.g., ISO 35001) for laboratory biosafety and biosecurity. Facilitating cross-sector collaboration to enhance biosecurity innovation and address emerging threats
Education, Training, and Workforce Development	Developing and delivering standardized educational materials and training programs on safe, secure, and responsible research. Hosting workshops and conferences to keep professionals updated on the latest biosafety and biosecurity practices. Creating certification and recertification programs for biosafety and biosecurity professionals
Environmental Risk Assessment	Conducting assessments to evaluate and mitigate potential ecological risks of biotechnologies. Developing guidelines for conducting environmental impact assessments for biotechnology. Monitoring and evaluating the long-term ecological impacts of biotechnological applications
Laboratory Animal Care and Use	Overseeing the humane and ethical care and use of animals at research facilities. Reviewing animal welfare assurances. Maintaining a registry of Institutional Animal Care and Use Committees
Biological Material Import and Transport	Reviewing applications for the import of infectious biological materials and vectors. Establishing secure and standardized procedures for the import and export of biological materials. Ensuring compliance with shipping protocols for biological materials and international regulations. Monitoring the transportation of high-risk biological materials to prevent unauthorized access or misuse

The agency would be responsible for all aspects of biosafety in the field and in the laboratory. The agency would establish clear standards for biological safety levels (e.g., BSL-2, BSL-3, BSL-4) that would integrate the guidance contained in the CDC and NIH’s *Biosafety in Microbiological and Biomedical Laboratories* manual and the *NIH Guidelines for Research Involving Recombinant or Synthetic Nucleic Acid Molecules*. The NBBA would have, and execute, the authority to create regulations, based on these standards, governing the design, construction, and certification of biocontainment facilities (United States Government Accountability Office, 2009; Lauer et al., 2023; National Institutes of Health, 2024). These biosafety standards would include comprehensive incident and emergency response protocols, incident response exercises, and training standards for laboratory personnel. The agency would also develop a national registry of all BSL-3 and BSL-4 facilities (Gillum et al., 2024). This registry would provide the basis for an inspection and audit system to ensure compliance with biosafety and biosecurity regulations and standards.

The proposed agency would have extensive responsibilities aimed at managing and improving biosafety and biosecurity policies and practices. This would include maintaining a centralized incident reporting system, operating a dedicated hotline for whistleblowers, and conducting investigations of biosafety and biosecurity incidents to gather lessons learned, develop preventative measures, identify gaps and weaknesses in biosafety and biosecurity standards, and uphold accountability. The agency would also conduct and sponsor empirically-based applied biosafety and biosecurity research (National Science and Technology Council, 2022; Casagrande, 2022) that would provide the basis for evidence-based biosafety and biosecurity standards and training requirements. This research program would produce a cadre of experienced biosafety and biosecurity researchers and allow the agency to optimize biosafety and biosecurity standards over time, and position the United States as a leader in global biosafety and biosecurity oversight (Johns Hopkins Center for Health Security, 2023). To avoid a real or perceived conflict of interest, the NBBA would not conduct or fund high-risk biological research. Instead, the NBBA would use extramural research grants to sponsor applied biosafety and biosecurity research in government, academic, or private facilities using less hazardous surrogates to ensure safety and compliance. The agency would identify ways to improve existing biosafety and biosecurity measures by identifying and eliminating ineffective policies and practices, or replacing them with more effective, evidence-based solutions. By continually refining standards based on the latest research and risk assessments, the agency would foster an environment of continual improvement and innovation in biosafety and biosecurity. The agency would develop criteria for when to add to or remove a pathogen from a regulated list (Lim and Popescu, 2024; Millett et al., 2023) and develop standards to reduce the risk of exposure to infectious diseases during biomedical and environmental sample collection and the handling of wild animals in the field (Cox et al., 2019; Aguilar-Setién et al., 2022).

The proposed agency would also develop and implement policies on the oversight of dual-use research, conduct risk assessments of emerging technologies, and develop policies to mitigate the risks posed by those technologies. A national system for oversight of dual-use research conducted by publicly and privately funded research institutions would be developed on the basis of the May 2024 Office of Science and Technology (OSTP) policy on dual-use research of concern and pathogens with enhanced pandemic potential (Office of Science and Technology Policy, 2024c; Office of Science and Technology Policy, 2024b). The agency would also oversee the implementation of the Department of Health and Human Services and OSTP nucleic acid synthesis screening policies and relevant provisions of the 30 October 2023 executive order on the safe and secure use of artificial intelligence to prevent the misuse of synthetic biology (Department of Health and Human Services, 2023; The White House, 2023; Office of Science and Technology Policy, 2024a). Conducting environmental impact assessments for biotechnological research and applications, such as those involving gene drives and genetically modified insects, would be crucial to mitigate potential risks to ecosystems (Evans and Palmer, 2018; Kuzma, 2020; Reynolds, 2020; Devos et al., 2022). The NBBA would also assess the risks and benefits of *in silico* experiments, synthetic biology, genome editing, and other emergent technologies, and identify options for ensuring that such technologies are developed and used in a safe, secure, and responsible manner (Carter et al., 2014; Anklam et al., 2022; Kuiken, 2023; Hunter, 2024).

The NBBA would standardize biosafety and biosecurity training and procedures, ensuring consistency across institutions and reducing the need for each organization to independently create their own training programs and safety protocols. The agency would establish minimum competency requirements for personnel in high-containment laboratories, recommend best practices for all biological research (Gillum et al., 2024), and develop biosecurity credentials for professionals working in high-containment laboratories and other sensitive areas (Moritz et al., 2020). By developing certification programs and requiring regular recertification to keep pace with evolving risks and technologies, the agency would ensure a standardized level of competence and awareness across the industry. The agency would also develop and disseminate awareness-raising, training, and educational materials to scientists, administrators, institutions, and companies subject to the oversight regarding dual-use research and nucleic acid screening. This approach would improve the overall quality, efficiency, and effectiveness of training and support documentation. Finally, the agency could serve as a forum for stakeholders to share information and exchange best practices on how to conduct life sciences research and biotechnology development safely, securely, and responsibly. The agency’s combined responsibility for biosafety and biosecurity standard-setting, applied biosafety and biosecurity research, and workforce development would allow the agency to continually refine standards based on the latest scientific research and risk assessments, disseminate them widely, and foster an environment of perpetual improvement and innovation in biosafety and biosecurity, thereby enhancing public health and safety.

The agency would collaborate with subject matter experts from the CDC, NIH, EPA, USDA, OSHA and other relevant organizations to provide current information on biosafety and biosecurity concerns related to infectious diseases, emerging threats, and evolving technologies. For example, the agency would coordinate with OSHA on how the Bloodborne Pathogens and forthcoming Infectious Diseases regulations impact biosafety and biosecurity. The agency would also collaborate with national security agencies to prevent bioterrorism and promote biosecurity innovation (Lentzos et al., 2020; Smith and Sandbrink, 2022). The agency would also conduct extensive awareness-raising, outreach, and engagement with stakeholders in academia and the private sector, as well as the general public, to solicit input on proposed policies, receive feedback on existing policies, and provide updates on recent and upcoming developments.

Lastly, international collaboration would be crucial to the NBBA’s efforts, aiming to strengthen and harmonize global standards (National Science Advisory Board for Biosecurity, 2023). NBBA would work with multilateral organizations, international networks, and coalitions of like-minded nations to build biosafety and biosecurity management capacity in partner nations and promote the adoption of international standards for biosafety and biosecurity management such as ISO 35001 and ISO/TS 5441:2024 (Koblentz and Lentzos, 2022). Given the proliferation of high containment labs, increase in high-risk research, growth in viral prospecting, the privatization and commercialization of life sciences research, and emergence of new methods, such as preprint servers, to distribute these breakthroughs faster than ever, global biosafety and biosecurity is only as strong as its weakest link (Koblentz and Lentzos, 2022). By ensuring the safe and secure advancement of biological research around the world, the agency would foster innovation while sustaining international competitiveness.

## 3 Conclusion

Establishing a unified biosafety and biosecurity agency would mark a pivotal advancement in the regulation of biological research in the United States. By consolidating existing oversight functions into a single entity, the NBBA would eliminate redundancies, streamline processes, and enhance overall efficiency and safety. This comprehensive approach would provide clear and consistent rules for researchers, institutions, and the public, promoting compliance and fostering an environment where scientific progress can thrive securely.

Addressing political and practical challenges is essential for the success of the new agency. Establishing the NBBA will require a comprehensive vision, detailed planning, and strong leadership. The fact that much of this new agency’s portfolio is not currently within the mission of any existing federal agency means that new resources—and not just the transfer of existing resources—will be required. Additionally, the strong political bias against creating new government agencies will pose significant obstacles, especially in areas where certain interest groups would prefer them to remain unregulated. On the other hand, should greater public demand for biosafety and biosecurity regulation arise suddenly, as in response to a deleterious biosafety incident, Congress may respond by enacting new authorities that are not as carefully crafted as the NBBA. Engaging stakeholders through public consultations, advisory committees, and open forums will be required to develop regulations, policies, and guidelines that are both comprehensive and inclusive and strike the appropriate balance between the need for safety and security and for scientific innovation. Collaboration with existing bodies such as the CDC, USDA, NIH, national security agencies, and international counterparts will help create a cohesive biosafety and biosecurity oversight system that addresses existing gaps and prepares for future challenges. By following this process, the agency can achieve high standards of biosafety and biosecurity, adapt to new challenges and opportunities, and support a vibrant life sciences research enterprise and robust bioeconomy.

The establishment of an agency dedicated to biosafety and biosecurity would significantly enhance national and global biosafety and biosecurity standards. By centralizing oversight, promoting education and workforce development, standardizing incident response reporting, and fostering international collaboration, the NBBA will ensure the safe and secure advancement of biological research. This agency will not only address the complexities of modern biotechnology with a robust regulatory framework but also support safe and efficient biotechnological innovations, thereby enhancing the nation’s bioindustrial capabilities and maintaining international competitiveness.

## References

[B1] Aguilar-Setién A. Aréchiga-Ceballos N. Balsamo G. A. Behrman A. J. Frank H. K. Fujimoto G. R. (2022). Biosafety practices when working with bats: a guide to field research considerations. Appl. Biosaf. 27 (3), 169–190. 10.1089/apb.2022.0019 Accessed July 31, 2024) 36196095 PMC9526472

[B2] Anklam E. Bahl M. I. Ball R. Beger R. D. Cohen J. Fitzpatrick S. (2022). Emerging technologies and their impact on regulatory science. Exp. Biol. Med. 247 (1), 1–75. 10.1177/15353702211052280 Accessed July 31, 2024) PMC8749227 34783606

[B3] Atlas R. M. Dando M. (2006). The dual-use dilemma for the life sciences: perspectives, conundrums, and global solutions. Biosecurity bioterrorism biodefense strategy, Pract. Sci. 4 (3), 276–286. 10.1089/bsp.2006.4.276 Accessed July 31, 2024) 16999588

[B4] Atlas R. M. Reppy J. (2005). Globalizing biosecurity. Biodefense Strategy, Pract. Sci. 3 (1), 51–60. 10.1089/bsp.2005.3.51 Accessed July 31, 2024) 15853455

[B5] Attal-Juncqua A. Dods G. Crain N. Diggans J. Dodds D. Evans S. (2023). Shaping the future US bioeconomy through safety, security, sustainability, and social responsibility. Trends Biotechnol. 42, 671, 673. 10.1016/j.tibtech.2023.11.015 Accessed 31 July 2024]. 38129216

[B6] Beck U. (1992). Risk society: towards a new modernity. London, United Kingdom: Sage.

[B7] Bory P. Crabu S. Morsello B. Tomasi M. Tosoni S. (2021). Rethinking the nexus between science, politics and society in the age of the SARS-CoV-2 pandemic. Tecnoscienza 12 (2), 140–187. 10.6092/issn.2038-3460/17546 Accessed July 31, 2024)

[B8] Carter S. R. Rodemeyer M. Garfinkel M. S. Friedman R. M. (2014). *Synthetic biology and the US biotechnology regulatory system: Challenges and options* (No. DOE-JCVI-SC0004872). Rockville, MD: J. Craig Venter Institute. Available at: https://www.jcvi.org/research/synthetic-biology-and-us-biotechnology-regulatory-system-challenges-and-options (Accessed July 31, 2024).

[B9] Casagrande R. (2022). Federal funding for biosafety research is critically needed. Washington, DC: Center for Strategic and International Studies CSIS. Available at: https://www.csis.org/analysis/federal-funding-biosafety-research-critically-needed (Accessed July 31, 2024)

[B10] Cox R. J. Nol P. Ellis C. K. Palmer M. V. (2019). Research with agricultural animals and wildlife. ILAR J. 60 (1), 66–73. 10.1093/ilar/ilz006 Accessed July 31, 2024) 31095682

[B11] Department of Health and Human Services (2023). Screening framework guidance for providers and users of synthetic nucleic acids. Available at: https://aspr.hhs.gov/legal/synna/Documents/SynNA-Guidance-2023.pdf (Accessed July 31, 2024).

[B12] Devos Y. Mumford J. D. Bonsall M. B. Camargo A. M. Firbank L. G. Glandorf D. C. (2022). Potential use of gene drive modified insects against disease vectors, agricultural pests and invasive species poses new challenges for risk assessment. Crit. Rev. Biotechnol. 42 (2), 254–270. 10.1080/07388551.2021.1933891 Accessed July 31, 2024) 34167401

[B13] DiEuliis D. Giordano J. (2022). The need for modernization of biosecurity in the post-COVID world. mSphere 7 (2), e0002522–22. 10.1128/msphere.00025-22 Accessed July 31, 2024) 35224977 PMC9044947

[B14] Eisner M. A. (2000). Regulatory politics in transition. Baltimore, MD: JHU Press.

[B15] Epa F. D. A. USDA (2017). Coordinated framework for the regulation of biotechnology. Available at: https://usbiotechnologyregulation.mrp.usda.gov/biotechnologygov/home (Accessed July 31, 2024).

[B16] Epstein G. (2023). Private-sector research could pose a pandemic risk. Here’s what to do about it. Bull. Atomic Sci. Available at: https://thebulletin.org/2023/02/private-sector-research-could-pose-a-pandemic-risk-heres-what-to-do-about-it/(Accessed July 31, 2024).

[B17] Epstein S. (1995). The construction of lay expertise: AIDS activism and the forging of credibility in the reform of clinical trials. Sci. Technol. and Hum. Values 20 (4), 408–437. 10.1177/016224399502000402 Accessed July 31, 2024) 11653331

[B18] Evans S. W. Beal J. Berger K. Bleijs D. A. Cagnetti A. Ceroni F. (2020). Embrace experimentation in biosecurity governance. Science 368 (6487), 138–140. 10.1126/science.aba2932 Accessed July 31, 2024) 32273459

[B19] Evans S. W. Palmer M. J. (2018). Anomaly handling and the politics of gene drives. J. Responsible Innovation 5 (Suppl. 1), S223–S242. 10.1080/23299460.2017.1407911 Accessed July 31, 2024)

[B20] Gillum D. Moritz R. Lim Y.-B. Vogel K. (2022). Charting a new course for biosafety in a changing world. Issues Sci. Technol. 23 May. Available at: https://issues.org/new-course-biosafety-prevent-pandemics-gillum-moritz-lim-vogel/(Accessed July 31, 2024).

[B21] Gillum D. R. Moritz R. L. Schwartz A. (2023). Effectively implementing biosecurity policies. Science 380 (6642), 251–252. 10.1126/science.adh5519 Accessed July 31, 2024) 37079682

[B22] Gillum D. R. Schwartz A. Albrecht R. A. Moritz R. L. (2024). Seven opportunities for effective biosafety and biosecurity governance. Health Secur. 22, 324–329. 10.1089/hs.2023.0189 Accessed July 31, 2024) 38608244

[B23] Greene D. Cerles A. Casagrande R. (2023) “Characterizing the private sector in human pathogen research,”. Health Security. 10.1089/hs.2024.0003 39393922

[B25] Gronvall G. K. (2013). H5N1: a case study for dual-use research. New York, NY: Council on Foreign Relations. Available at: https://cdn.cfr.org/sites/default/files/pdf/2013/05/WP_Dual_Use_Research.pdf (Accessed July 31, 2024).

[B26] Hodgson A. Maxon M. E. Alper J. (2022). The US bioeconomy: charting a course for a resilient and competitive future. Ind. Biotechnol. 18 (3), 115–136. 10.1089/ind.2022.29283.aho Accessed July 31, 2024)

[B27] Hunter P. (2024). Security challenges by AI-assisted protein design: the ability to design proteins *in silico* could pose a new threat for biosecurity and biosafety. EMBO Rep. 25 (5), 2168–2171. 10.1038/s44319-024-00124-7 Accessed July 31, 2024) 38532127 PMC11094011

[B28] Jasanoff S. (2006). Transparency in public science purposes, reasons, limits. Law and Contemp. Problems 69, 21. Available at: https://scholarship.law.duke.edu/cgi/viewcontent.cgi?referer=&httpsredir=1&article=1385&context=lcp (Accessed July 31, 2024).

[B29] Johns Hopkins Center for Health Security (2023) “Building strong biosafety and biosecurity into the expanding US bioeconomy,” in Meeting report on a 10 january 2023 public-private roundtable organized by the johns Hopkins center for health security. Washington, D.C.: Johns Hopkins Center for Health Security. Available at: https://www.centerforhealthsecurity.org/sites/default/files/2023-04/20230228-biosafety-and-biosecurity.pdf (Accessed July 31, 2024).

[B30] Kanabrocki J. (2011). Interactions between biocontainment laboratories and their communities: a successful work in progress. Trends Microbiol. 19 (5), 209–210. 10.1016/j.tim.2011.01.008 Accessed July 31, 2024) 21353566

[B31] Kaplan L. R. Farooque M. Sarewitz D. Tomblin D. (2021). Designing participatory technology assessments: a reflexive method for advancing the public role in science policy decision-making. Technol. Forecast. Soc. Change 171, 120974. 10.1016/j.techfore.2021.120974 Accessed July 31, 2024)

[B32] Kelle A. (2013). Beyond patchwork precaution in the dual-use governance of synthetic biology. Sci. Eng. Ethics 19, 1121–1139. 10.1007/s11948-012-9365-8 Accessed July 31, 2024) 22535577 PMC3735959

[B33] Koblentz G. D. (2014). Dual-use research as a wicked problem. Front. Public Health 2, 113. 10.3389/fpubh.2014.00113 Accessed July 31, 2024) 25140299 PMC4121526

[B34] Koblentz G. D. Casagrande R. (2023). Biology is dangerously outpacing policy. New York Times. Available at: https://www.nytimes.com/2023/02/20/opinion/biology-is-dangerously-outpacing-policy.html (Accessed July 31, 2024)

[B35] Koblentz G. D. Lentzos F. (2022). A plan B to strengthen biosafety and biosecurity. Think. Glob. Health. Available at: https://www.thinkglobalhealth.org/article/plan-b-strengthen-biosafety-and-biosecurity (Accessed July 31, 2024).

[B36] Kojima K. Booth C. M. Summermatter K. Bennett A. Heisz M. Blacksell S. D. (2018). Risk-based reboot for global lab biosafety. Science 360 (6386), 260–262. 10.1126/science.aar2231 Accessed July 31, 2024) 29674576

[B37] Kuhlau F. Eriksson S. Evers K. Höglund A. T. (2008). Taking due care: moral obligations in dual use research. Bioethics 22 (9), 477–487. 10.1111/j.1467-8519.2008.00695.x Accessed July 31, 2024) 18959730

[B38] Kuiken T. (2023). Artificial intelligence in the biological sciences: uses, safety, security, and oversight. Congr. Res. Serv. (CRS) Rep. Issue Briefs. Available at: https://crsreports.congress.gov/product/pdf/R/R47849 (Accessed July 31, 2024).

[B39] Kuzma J. (2020). Engineered gene drives: ecological, environmental, and societal concerns. GMOs Implic. Biodivers. conservation Ecol. Process., 371–399. 10.1007/978-3-030-53183-6_17 Accessed July 31, 2024)

[B40] Lauer E. Kim K. N. Dettmann R. A. Fleming A. E. Powell G. L. Rice A. D. (2023). Lessons learned from the design, construction, and commissioning of a retrofitted arthropod containment level 3 insectary. Am. J. Trop. Med. Hyg. 109 (1), 126–133. 10.4269/ajtmh.22-0790 Accessed July 31, 2024) 37188338 PMC10324019

[B41] Le Duc J. W. Weaver S. C. (2024). A bottom-up approach to biosecurity. Health Secur. 22, 343–346. 10.1089/hs.2023.0170 Accessed July 31, 2024) 38717837

[B42] Lentzos F. Goodman M. S. Wilson J. M. (2020). Health security intelligence: engaging across disciplines and sectors. Intell. Natl. Secur. 35 (4), 465–476. 10.1080/02684527.2020.1750166 Accessed July 31, 2024)

[B43] Lentzos F. Koblentz G. D. Rodgers J. (2022). The urgent need for an overhaul of global biorisk management. CTC Sentin. Available at: https://ctc.westpoint.edu/the-urgent-need-for-an-overhaul-of-global-biorisk-management/(Accessed July 31, 2024).

[B44] Lim Y. B. Gillum D. Vogel K. (2021). Twenty years after the patriot Act, what is the future of biosecurity? Issues Sci. Technol. Available at: https://issues.org/biosecurity-20-years-after-patriot-act-lim-gillum-vogel/(Accessed July 31, 2024).

[B45] Lim Y. B. Popescu S. (2024). Exploring list-based approaches and potential threat agnostic applications in US biodefense and public health—toward a hybrid approach. Health Secur. 22 (2), 146–155. Available at:. 10.1089/hs.2023.0098 Accessed July 31, 2024) 38546510

[B46] Lin A. C. (2010). Technology assessment 2.0: revamping our approach to emerging technologies. Brooklyn Law Rev. 76, 1309. Available at: https://brooklynworks.brooklaw.edu/cgi/viewcontent.cgi?article=1178&context=blr (Accessed July 31, 2024).

[B47] Miller C. A. Bennett I. (2008). Thinking longer term about technology: is there value in science fiction-inspired approaches to constructing futures? Sci. Public Policy 35 (8), 597–606. Available at:. 10.3152/030234208X370666 Accessed July 31, 2024)

[B48] Miller S. Selgelid M. J. (2007). Ethical and philosophical consideration of the dual-use dilemma in the biological sciences. Sci. Eng. ethics 13, 523–580. 10.1007/s11948-007-9043-4 Accessed July 31, 2024) 18060518 PMC7089176

[B49] Millett P. Alexanian T. Brink K. R. Carter S. R. Diggans J. Palmer M. J. (2023). Beyond biosecurity by taxonomic lists: lessons, challenges, and opportunities. Health Secur. 21 (6), 521–529. 10.1089/hs.2022.0109 Accessed July 31, 2024) 37856148 PMC10733751

[B50] Moritz R. L. Berger K. M. Owen B. R. Gillum D. R. (2020). Promoting biosecurity by professionalizing biosecurity. Science 367 (6480), 856–858. 10.1126/science.aba0376 Accessed July 31, 2024) 32079762

[B51] National Academies of Sciences, Engineering, and Medicine (2017). Dual Use Research of Concern in the Life Sciences: Current Issues and Controversies. Washington, DC: The National Academies Press. 10.17226/24761 29001489

[B52] National Institutes of Health (2024). Design requirements manual. Available at: https://orf.od.nih.gov/TechnicalResources/Pages/DesignRequirementsManual2016.aspx (Accessed July 31, 2024).

[B53] National Science Advisory Board for Biosecurity (2023). Proposed biosecurity oversight framework for the future of science. Washington, D.C.: National Institutes of Health. Available at: https://osp.od.nih.gov/wp-content/uploads/2023/03/NSABB-Final-Report-Proposed-Biosecurity-Oversight-Framework-for-the-Future-of-Science.pdf (Accessed July 31, 2024).

[B54] National Science and Technology Council (2022). Evidence-based laboratory biorisk management science and technology roadmap. Available at: https://www.whitehouse.gov/wp-content/uploads/2022/04/04-2022-NSTC-ST-Biorisk-Research-Roadmap_FINAL.pdf (Accessed July 31, 2024).

[B55] Nie J. B. (2020). In the shadow of biological warfare: conspiracy theories on the origins of COVID-19 and enhancing global governance of biosafety as a matter of urgency. J. Bioethical Inq. 17 (4), 567–574. 10.1007/s11673-020-10025-8 Accessed July 31, 2024) PMC7445685 32840850

[B56] Office of Science Technology and Policy (2024a). Framework on nucleic acid synthesis screening. Available at: https://www.whitehouse.gov/wp-content/uploads/2024/04/Nucleic-Acid_Synthesis_Screening_Framework.pdf (Accessed July 31, 2024).

[B57] Office of Science Technology and Policy (2024b). Implementation guidance for the United States government policy for oversight of dual use research of concern and pathogens with enhanced pandemic potential. Available at: https://www.whitehouse.gov/wp-content/uploads/2024/05/USG-DURC-PEPP-Implementation-Guidance.pdf (Accessed July 31, 2024).

[B58] Office of Science Technology and Policy (2024c). United States government policy for oversight of dual use research of concern and pathogens with enhanced pandemic potential. Available at: https://www.whitehouse.gov/wp-content/uploads/2024/05/USG-Policy-for-Oversight-of-DURC-and-PEPP.pdf (Accessed July 31, 2024).

[B59] Quinlan M. M. Smith J. Layton R. Keese P. Agbagala M. L. U. Palacpac M. B. (2016). Experiences in engaging the public on biotechnology advances and regulation. Front. Bioeng. Biotechnol. 4, 3. 10.3389/fbioe.2016.00003 Accessed July 31, 2024) 26870726 PMC4735352

[B60] Reynolds J. L. (2020). Governing new biotechnologies for biodiversity conservation: gene drives, international law, and emerging politics. Glob. Environ. Polit. 20 (3), 28–48. 10.1162/glep_a_00567 Accessed July 31, 2024)

[B61] Richardson L. C. Connell N. D. Lewis S. M. Pauwels E. Murch R. S. (2019). Cyberbiosecurity: a call for cooperation in a new threat landscape. Front. Bioeng. Biotechnol. 7, 99. 10.3389/fbioe.2019.00099 Accessed July 31, 2024) 31245363 PMC6562220

[B62] Ritterson R. Kingston L. Fleming A. E. Lauer E. Dettmann R. A. Casagrande R. (2022). A call for a national agency for biorisk management. Health Secur. 20 (2), 187–191. 10.1089/hs.2021.0163 Accessed July 31, 2024) 35302871

[B63] Smith J. A. Sandbrink J. B. (2022). Biosecurity in an age of open science. PLoS Biol. 20 (4), e3001600. 10.1371/journal.pbio.3001600 Accessed July 31, 2024) 35421093 PMC9009689

[B64] The White House (2021). Presidential memorandum on United States government-supported research and development national security policy. Available at: https://trumpwhitehouse.archives.gov/presidential-actions/presidential-memorandum-united-states-government-supported-research-development-national-security-policy/(Accessed July 31, 2024).

[B65] The White House (2022). Executive order on advancing biotechnology and biomanufacturing innovation for a sustainable, safe, and secure American bioeconomy. Available at: https://www.whitehouse.gov/briefing-room/presidential-actions/2022/09/12/executive-order-on-advancing-biotechnology-and-biomanufacturing-innovation-for-a-sustainable-safe-and-secure-american-bioeconomy/(Accessed July 31, 2024)

[B66] The White House (2023). Executive Order Safe, Secure, Trust. Dev. Use Artif. Intell. Available at: https://www.whitehouse.gov/briefing-room/presidential-actions/2023/10/30/executive-order-on-the-safe-secure-and-trustworthy-development-and-use-of-artificial-intelligence/(Accessed July 31, 2024)

[B67] United States Government Accountability Office (2009). High-containment laboratories: national strategy for oversight is needed. GAO-09-574. Washington, D.C.: U.S. Government Accountability Office. Available at: https://www.gao.gov/assets/gao-09-574.pdf (Accessed July 31, 2024)

[B68] United States Government Accountability Office (2017). High containment laboratories: coordinated actions needed to enhance the Select agent program’s oversight of hazardous pathogens. Washington, D.C.: U: S. Government Accountability Office. Available at: https://www.gao.gov/assets/gao-18-145.pdf (Accessed August 8, 2024).

[B69] Vallas S. P. Kleinman D. L. (2008). Contradiction, convergence and the knowledge economy: the confluence of academic and commercial biotechnology. Socio-economic Rev. 6 (2), 283–311. 10.1093/ser/mwl035 Accessed July 31, 2024)

[B70] Vogel K. M. (2012). Phantom menace or looming danger? a new framework for assessing bioweapons threats. Baltimore, MD: JHU Press.

[B71] Vogel K. M. (2013). Expert knowledge in intelligence assessments: bird flu and bioterrorism. Int. Secur. 38 (3), 39–71. 10.1162/ISEC_a_00150 Accessed July 31, 2024)

[B72] Warmbrod K. L. Trotochaud M. Gronvall G. K. (2020). Shaping the US bioeconomy for future economic development and sustainability. Health Secur. 18 (4), 265–266. 10.1089/hs.2020.0122 Accessed July 31, 2024) 32816591

[B73] Wynne B. (1992). Uncertainty and environmental learning: reconceiving science and policy in the preventive paradigm. Glob. Environ. Change 2 (2), 111–127. 10.1016/0959-3780(92)90017-2 Accessed July 31, 2024)

[B74] You E. (2017). Safeguarding the bioeconomy: U.S. Opportunities and challenges. Testimony U.S.-China Econ. Secur. Rev. Comm. Available at: https://www.ehidc.org/resources/safeguarding-bioeconomy-us-opportunities-and-challenges (Accessed July 31, 2024).

